# Characteristics and Management of a Cohort of Patients with Asymmetric Thyroid Eye Disease and Unilateral Dysthyroid Optic Neuropathy Undergoing Unilateral Orbital Decompression Surgery

**DOI:** 10.3390/jcm15218726

**Published:** 2026-11-08

**Authors:** Sandra Rezar-Dreindl, Lorenz Wassermann, Johannes Engelmann, Thomas Neumayer, Stefan Sacu, Eva Stifter

**Affiliations:** 1 Department of Ophthalmology and Optometry, Medical University of Vienna, 1090 Vienna, Austria; 2 Department of Oral and Maxillofacial Surgery, Medical University of Vienna, 1090 Vienna, Austria

**Keywords:** asymmetric thyroid eye disease, unilateral dysthyroid optic neuropathy, orbital decompression surgery, intravenous corticosteroids

## Abstract

**Background/Objectives**: The aim was to describe the clinical characteristics and report the long-term outcomes of a cohort of patients with asymmetric thyroid eye disease (TED), including a subgroup with unilateral dysthyroid optic neuropathy (DON), treated with unilateral orbital decompression surgery alone or in combination with intravenous (IV) methylprednisolone. **Methods:** This retrospective study included 48 patients with asymmetric TED who all underwent orbital decompression with or without IV corticosteroid therapy between 2006 and 2020 at the Department of Ophthalmology and Optometry and Oral and Maxillofacial Surgery of the Medical University of Vienna, Austria. **Results:** The mean follow-up was 4.6 ± 2.6 years. Overall, 52% of the patients initially received IV corticosteroids followed by orbital decompression, and 73% underwent three-wall decompression. Mean visual acuity (VA) was 0.1 ± 0.5 logMAR at baseline and 0.1 ± 0.5 logMAR at final follow-up (*p* = 0.4). The mean reduction in proptosis was 4 ± 3 mm. Unilateral DON was diagnosed in 50% of the patients. In this subgroup, 67% were initially treated with IV corticosteroids and 79% underwent three-wall decompression. Mean reduction in proptosis was 5 mm. The final VA of the affected eye was <0.3 logMAR in 88% (21/24) of the patients with unilateral DON. **Conclusions:** Patients with asymmetric TED were predominantly treated with IV corticosteroids and received three-wall orbital decompression. Visual function could be maintained in a high number of cases with asymmetric TED and patients with unilateral DON.

## 1. Introduction

Thyroid eye disease (TED), also known as Graves’ orbitopathy or thyroid-associated orbitopathy, has an estimated prevalence of 10/10,000 individuals in Europe [1]. In patients with Graves’ disease, clinical changes including lid retraction, exophthalmos and diplopia can develop during disease progression in up to 50% of patients representing TED [1,2,3,4,5]. In most cases involvement is bilateral and symmetric; however, in some cases the disease can present unilaterally or asymmetrically. The reasons for unilateral and asymmetric presentation of TED remain poorly understood.

The prevalence of unilateral disease varies widely in the literature and is reported to be from 4.5% up to 14%. Also the prevalence of asymmetric disease varies from 9 to 34% [6]. Several risk factors have been associated with unilateral or asymmetric TED, including a shorter duration of symptoms, euthyroid or hypothyroid status, sex and age [7,8]. Nevertheless, there is no standard definition for unilateral or highly asymmetric TED. Most studies define unilateral disease as an interocular difference in proptosis of greater than 2 mm, or of ≥2 mm accompanied by symptoms and signs of TED (such as retrobulbar pain, anterior segment findings or soft-tissue involvement), in only one eye, with absent or milder signs in the fellow eye. Radiological definitions based on the right-to-left ratio have also been proposed [8,9,10].

The diagnosis of asymmetric TED can be challenging because of its atypical presentation of disease and potential differential diagnoses such as orbital tumours. To date, knowledge of the clinical course of unilateral or asymmetric TED remains limited, and no guidelines are available for its management. Within this analysis, the characteristics and course of disease of only unilateral TED cases who were all treated with orbital decompression surgery were analyzed in order to gain more information about this untypical disease. The aim of the study was to evaluate the clinical characteristics of patients with asymmetric TED and unilateral DON, as well as their management strategies and long-term outcomes.

## 2. Materials and Methods

### 2.1. Study Design and Patients

**Figure 1:**
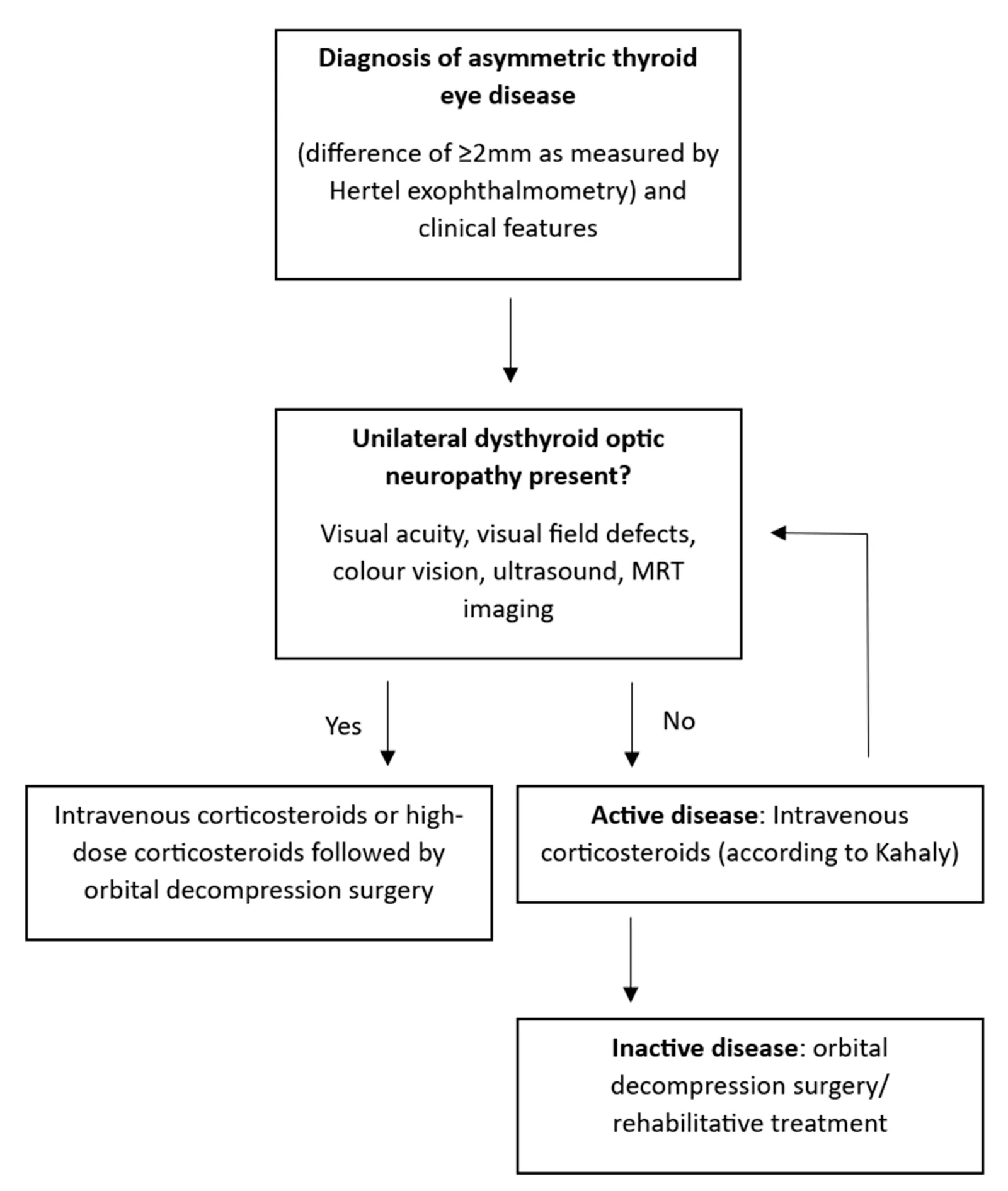
Treatment pathways of patients with asymmetric TED or unilateral DON.

This was a retrospective analysis of patients with asymmetric TED or unilateral DON. All asymmetric TED patients were evaluated and treated at the Department of Ophthalmology and Optometry and the Department of Oral and Maxillofacial Surgery of the Medical University of Vienna between 2006 and 2020. Patients were all treated with orbital decompression surgery and/or received IV methylprednisolone. Patients with a follow-up of less than one year were excluded. In our evaluation asymmetric disease was defined as an interocular difference in exophthalmos of ≥2 mm as measured by Hertel exophthalmometry. Patients with signs of soft-tissue involvement, mild motility restriction or mild lid retraction were not excluded. DON was diagnosed according to the criteria of the European Group on Graves’ Orbitopathy (EUGOGO) [11]. Diagnosis of unilateral DON was based on decreased VA, impaired colour vision, a relative afferent pupillary defect, optic disc swelling and/or visual field defects, supported by ultrasonography or MRT imaging. Interdisciplinary management of the patients was carried out together by the Department of Endocrinology and the Department of Oral-Maxillofacial Surgery. When treatment with corticosteroids was indicated for active inflammation, it was administered in accordance with Kahaly et al. [12] and the EUGOGO recommendations [11,13]. The treatment pathways are shown in Figure 1.

### 2.2. Surgical Technique and Navigation Device

The surgical technique (removal of one, two or three bony walls) was chosen based on the surgeon’s preference and expertise [14]. The surgical approach was a two- or three-wall decompression with orbital fat resection when necessary, depending on the clinical and aesthetical situation and on ophthalmic parameters, including loss of vision, degree of proptosis and lack of eyelid closure. In cases of high degrees of proptosis and lack of eyelid closure, a three-wall decompression was preferred. Two-wall decompression (orbital floor and medial wall removal) was preferred for less pronounced proptosis. Two-wall decompression with emphasis on the posterior part can be used to treat DON [15].

Prior to orbital decompression a computer tomography (CT) evaluation for planning of the facial skeleton was performed for all patients. The Digital Imaging and Communications in Medicine (DICOM) data were transferred to the navigation workstation. The decision to use the navigation system depended on the individual patient’s anatomy and the surgeon’s experience. Surgical technique and navigation techniques have been described elsewhere in detail [16].

### 2.3. Statistical Analysis

Descriptive statistics and frequency distributions were calculated for all variables. The aim of the analysis was to evaluate the management of patients with asymmetric TED or unilateral DON treated with orbital decompression surgery alone or with additional IV methylprednisolone. Baseline variables were analyzed for all the patients. The course of VA over time, exophthalmos and intraocular pressure (IOP) over time were reported. Information regarding treatment was described. Changes in VA over time were assessed using two-sided paired *t*-tests. For comparisons between two groups (patients with and without DON), independent *t*-tests were used. The level of statistical significance was set to *p* <0.05.

## 3. Results

### 3.1. Patient Characteristics

The characteristics of the 48 patients with asymmetric TED are summarized in Table 1. The mean age was 50 ± 13 years (mean ± standard deviation; range, 30–79 years), and the mean follow-up was 4.6 ± 2.6 years (median, 3.8 years; range, 1–11.6 years). Twenty-four patients (50%) had undergone thyroidectomy; in 31% (15/48), thyroidectomy had been performed within one year before the onset of TED. Three patients did not receive oral corticosteroid prophylaxis after thyroidectomy.

Ocular comorbidities potentially limiting VA included choroidal neovascularization secondary to age-related macular degeneration (AMD) in one patient (2%), AMD without neovascularization in two patients (4%) and macular hemorrhage in one patient (2%). Systemic comorbidities included arterial hypertension (2%, 1/48), diabetes mellitus (4%, 2/48) and a history of stroke (2%, 1/48). Regarding smoking status, 44% (21/48) were current smokers, 35% (17/48) former smokers and 13% (6/48) never smokers; smoking status was unknown in 8% (4/48).

### 3.2. Clinical Symptoms

Diplopia was the most frequently symptom reported at presentation (44%, 21/48), followed by increase in exophthalmos (42%, 20/48), reduced or blurred vision or impaired colour vision (15%, 7/48), and dry-eye symptoms (10%, 5/48). Lid lag was present in three patients (6%) at first presentation. The mean clinical activity score (CAS) at presentation was 3 ± 2/2 ± 2 (median: 3/1; affected eye/fellow eye). CAS was not documented at initial presentation in 14 patients (29%). A CAS > 3 was documented in 19% (9/48) of patients.

### 3.3. Ocular Motility and Proptosis Before Treatment

At initial presentation, 71% (34/48) of patients had restricted upgaze. Upgaze restriction was the only motility finding in 17% (8/48) and was combined with restricted abduction in 15% (7/48), with restricted adduction in 8% (4/48), and with restricted adduction in a further 15% (7/48). Isolated abduction restriction was found in 6% (3/48), restriction in all directions of gaze was found in 8% (4/48), and 10% (5/48) had no motility restriction. The vertical palpebral fissure height was 11 ± 2.4 mm in the affected eye and 10 ± 2.3 mm in the fellow eye (median, 10 and 11 mm; range, 7–16 and 7–19 mm, respectively).

Mean proptosis was 23 ± 3 mm/21 ± 4 mm in the affected eye/fellow eye (median: 23/20 mm; ranges 16–32/15–31 mm). An interocular difference of more than 5 mm was present in 13% (6/48) of patients. Proptosis was ≤25 mm in 77% (37/48), 26–30 mm in 21% (10/48) and 31 mm in 2% (1/48). The ultrasonographic superonasal muscle index (SNI) was 6.8 ± 0.5 (median, 6.8) overall, 6.7 ± 1.4 in the affected eye and 6.2 ± 0.8 in the fellow eye (median, 6.8 and 6.3, respectively). Before treatment, mean VA was 0.1 ± 0.5 logMAR in the affected eye and 0.05 ± 0.5 logMAR in the fellow eye (median, 0.05 and 0.0 logMAR, respectively).

### 3.4. Treatment and Outcomes

All patients underwent orbital decompression, either alone or after IV corticosteroid therapy. Following the diagnosis of asymmetric TED, 52% (25/48) of patients were initially treated with IV methylprednisolone, administered either as 500 mg weekly for 6 weeks followed by 250 mg weekly for 6 weeks, or as high-dose pulse therapy of 500 mg daily on 3 consecutive days.

In all patients, orbital decompression surgery was done. Surgery was done after a mean of 7.5 ± 11.7 months after initiation of corticosteroid therapy. Three-wall decompression was performed in 73% (35/48) and two-wall decompression in 27% (13/48) of patients. Indications for orbital decompression included dysthyroid optic neuropathy in 50% (*n* = 24), severe proptosis in 40% (*n* = 19), exposure keratopathy or lagophthalmos in 6% (*n* = 3) and a rehabilitative reason (strabismus or hypo/hypertropia) in 4% (*n* = 2).

At the last follow-up, mean VA was 0.05 ± 0.5 logMAR in the affected eye and 0.1 ± 0.5 logMAR in the fellow eye (median, 0.05 logMAR in both eyes). The final VA of the affected eye was ≤0.3 logMAR in 90% (43/48) of patients. Three patients (6%) had a final VA of ≥0.5 logMAR. These patients presented with reduced VA at initial presentation of 1.6, 0.8 and 0.3, respectively. One patient with final VA ≥ 0.5 had additional trauma of the affected eye with secondary cataract and one patient developed severe corneal ulceration due to incomplete lid closure, and one patient had additional macular hemorrhage on the affected eye. Two patients (4%) had a VA between 0.3 and 0.5 logMAR. VA was preserved (change within ±5 letters) or improved in 85% (41/48) of patients and decreased by more than five letters in 15% (7/48). In the latter patients, mean VA declined from 0.1 ± 0.7 logMAR at presentation to 0.3 ± 0.7 logMAR at final follow-up. The outcomes are summarized in Table 2. In the fellow eye, 85% (41/48) of patients had a final VA of ≤0.3 logMAR, and 15% (7/48) had a final VA of ≥0.4 logMAR. At the last follow-up, the mean CAS of the fellow eye was 1 ± 1 and mean exophthalmos was 21 ± 4 mm (median, 20 mm).

Ocular hypertension, defined as IOP ≥ 25 mmHg, occurred in 19% (9/48) of patients, with a maximum documented IOP of 45 mmHg during follow-up. All of these patients were treated with topical IOP-lowering medication.

### 3.5. Long-Term Follow-Up

After orbital decompression, one patient (2%) required prism correction, and diplopia persisted in up- or downgaze in 6% (3/48). In total, 31% of patients (15/48) showed no restriction in motility or only mild restriction at final follow-up. A total of 13% (6/48) had strabismus, all of them with an angle of deviation < 20 prism dioptres. Compensatory head posture between 5 and 10° was observed in 10% (5/48) and between 10 and 15° in 4% (2/48) of the patients. In total, 15% (7/48) of the patients had hypotropia and in 8% (4/48) upgaze was considerably restricted at the last follow-up examination.

Rehabilitative surgery (eyelid or strabismus surgery) was performed in 63% (30/48) after a mean of 10 months after the orbital decompression surgery. In 10% (5/48) of the patients, a second surgery became necessary. In total, 29% (14/48) of the patients had strabismus surgery, and 29% (14/48) had strabismus and lid surgery; in 4% (2/48) only lid surgery was performed.

### 3.6. Patients with Unilateral DON

In this analysis 50% (24/48) of the patients had a diagnosis of unilateral DON. Diagnosis was based on decreased VA, impaired colour vision, a relative afferent pupillary defect, optic disc swelling and/or visual field defects, supported by ultrasonography or MRT imaging. Reduced VA was present in 63% (*n* = 15) of all DON patients. Concentric narrowing of the visual field was evident in 46% (*n* = 11) and impaired colour vision in 42% (*n* = 10). Diagnostic tests included visual field testing and/or MRT (apical crowding) and measurement of extraocular muscle thickness (SNI) via ultrasound. The mean age of the patients was 55 ± 11. Their characteristics at initial presentation are shown in Table 3.

In total, 29% (7/24) of patients had thyroidectomy before diagnosis of unilateral DON. Three of them received thyroidectomy within 1 year before diagnosis of DON. A total of 13% (3/24) of the patients were previously treated with radioactive iodine.

Diplopia was the most commonly reported symptom at first examination (58%, 14/24). Increase in exophthalmos was noticed in 25% (6/24). In total, 29% of patients reported reduced or blurred vision or reduced colour vision (7/24), and 8% (2/24) mentioned sicca symptoms. The mean documented clinical activity score (CAS) at initial presentation was 3 ± 2. Mean proptosis was 24 ± 3 mm in the affected eye (median: 23; range 19–30). Measurement of the SNI was 7.1 ± 0.5 (median: 7.0).

Following diagnosis, 67% (16/24) of patients with DON were initially treated with IV methylprednisolone using one of the regimens described above. Two-wall decompression was performed in 21% (5/24) and three-wall decompression in 79% (19/24). At the last follow-up, mean proptosis of the affected eye was 19 ± 3 mm (median, 19 mm; range, 13–24 mm).

VA was 0.1 ± 0.5 (median 0.2) at initial presentation and was 0.2 ± 0.5 (median: 0.2) at final follow-up in the affected eye (*p* = 0.4). At the time of diagnosis of DON, 29% of patients had reduced visual acuity > 0.3 (*n* = 7), and 29% reduced visual acuity between 0.3 and 0.1; in 42%, visual acuity was not or was only slightly reduced (0.05–0.0 logMAR). At final follow-up, 13% of patients had reduced VA > 0.3 (*n* = 3), and 29% reduced visual acuity between 0.3 and 0.1; in 58%, visual acuity was not or was only slightly reduced (0.05–0.0 logMAR). In 88% (21/24) of the patients, final VA of the affected eye was ≤0.3 logMAR. In 21% visual acuity decreased in comparison to baseline.

Rehabilitative eyelid and/or strabismus surgery was required in 86% (21/24) of patients a mean of 9 months after decompression.

### 3.7. Subgroup Analysis: Patients with and Without DON

Among patients without DON, 67% (*n* = 16) were female and 33% (*n* = 8) were male, compared with 50% (*n* = 12) female and 50% (*n* = 12) male patients in the DON group. In the group without DON, 33% had two-wall decompression and 67% three-wall decompression. In comparison 79% of the patients in the DON group were treated with three-wall decompression and only 21% with two-wall decompression (*p* = 0.05). In patients with DON, the mean time from diagnosis to surgical decompression was 2.5 ± 1.8 months (*p* < 0.01). In comparison to patients without a diagnosis of DON, surgical decompression was done after a mean of 15 ± 11.8 months. In total, 42% (*n* = 10) of the patients without DON were initially treated with IV steroids and 67% (*n* = 16) of the group of patients with DON. Rehabilitative surgery was necessary in 46% (*n* = 11) in the group of patients without DON and in 86% (*n* = 21) of patients with DON. Table 4 shows the differences between patients with or without DON.

## 4. Discussion

Asymmetric TED and unilateral DON are uncommon, and their diagnosis can be challenging. The reasons for unilateral or asymmetric manifestation of TED remain unclear. Although several studies have compared unilateral with bilateral TED, none has specifically addressed patients with asymmetric TED or unilateral DON treated with orbital decompression with or without IV methylprednisolone. The present study describes the clinical characteristics of patients with asymmetric TED and of the subgroup with unilateral DON, and reports long-term outcomes of a treatment strategy consisting of orbital decompression with or without preceding IV methylprednisolone.

The lack of a standard definition of asymmetric TED limits the comparability of existing studies. Most studies define unilateral disease as an interocular difference in proptosis of >2 mm, or of ≥2 mm together with symptoms and signs of TED (including retrobulbar pain, anterior segment findings or soft-tissue involvement), confined to one eye or with fewer signs in the fellow eye [8,9,10]. In the present study, asymmetric disease was defined as an interocular difference in exophthalmos of ≥2 mm. Unilateral DON was defined as TED with reduced visual acuity, defects on automated perimetry, impaired colour vision, an SNI ≥ 7 on standardized ultrasonography, documented optic nerve compression on MRI/CT, optic disc swelling or elevated IOP [11]. A standardized definition of unilateral/asymmetric TED is needed to allow for better comparison between studies of this atypical presentation and to improve patient management.

Some risk factors have been identified which are associated with unilateral/asymmetric TED. One study reported no difference in age or sex; however, the interval between the onset of thyroid disease and development of thyroid eye disease was reported to be shorter [6]. In our cohort, the mean age was 50 ± 13 years; 42% of patients were male and 58% female. A mean age of 44 years has been reported for unilateral disease, and even younger ages in another series, whereas a study comparing asymmetric with symmetric disease reported a mean age of 50.9 years, which is consistent with our findings [9,17,18]. The wide range of reported ages may reflect the heterogeneity of the disease and differences in the ethnic composition of the study populations. In our cohort, diplopia (49%) and upgaze restriction (71%) were common findings, and diplopia was also the most frequent presenting symptom in patients with unilateral DON. In contrast, eyelid and periocular swelling were the most common presenting symptoms in patients with bilateral DON, although upgaze restriction was likewise the principal clinical finding in that population [16].

The treatment options are the same for bilateral or unilateral/asymmetric disease [13,19,20,21]. In our cohort, 52% of patients were initially treated with IV corticosteroids followed by orbital decompression. Three-wall decompression was performed in 73% and two-wall decompression in 27%, resulting in a mean reduction in proptosis of 4 ± 3 mm. No studies exist focusing on only unilateral/asymmetric TED/DON cases and treatment with corticosteroids and/or orbital decompression surgery. In a previous study of bilateral DON by our group, most patients underwent three-wall decompression with or without prior corticosteroids, resulting in a reduction in proptosis of 4–5 mm [16]. Previous studies have reported a mean reduction in exophthalmos of 3–5 mm, which is consistent with our findings [22,23], with greater reductions after three-wall than after two-wall decompression [23,24]. The greatest reduction was observed in patients with DON (5 mm), which may be explained by the fact that only five patients in this group underwent two-wall decompression. Surgical techniques and approaches for patients with TED vary and an individual management strategy must be used depending on the clinical presentation of the patient.

Following decompression surgery only 2% required prism correction, and 6% had diplopia only in up- or downgaze. In total, 13% had only mild strabismus with an angle of deviation < 20 prism dioptres. Rehabilitative surgery was performed in 63%. In 10% a second surgery was necessary for strabismus correction. Functional parameter remained stable during follow-up. Differences were observed regarding visual acuity change in patients with DON and TED. Differences in visual outcome were observed between patients with and without DON: VA decreased in 15% of the overall cohort and in 21% of the DON subgroup, underlining the sight-threatening nature of DON and the need for early diagnosis and specific management to preserve visual function in these patients. Unilateral TED has been reported to be more severe than bilateral disease. In a low number of patients, visual acuity was reduced in comparison to baseline. Studies reported unilateral TED cases to be more severe when compared to bilateral cases. However, in our cohort of patients, VA was reported to be maintained (0.1 ± 0.5 logMAR at initial presentation and 0.1 ± 0.5 logMAR at final follow-up). In 15% of patients, visual acuity decreased in comparison to baseline.

Our data showed that treatment initiation was mostly done with corticosteroids and in 71%, three-wall decompression surgery followed after a mean of 7.5 months. According to the EUGOGO guidelines, treatment with IV glucocorticoids for active TED and DON was recommended in 2016. After a reduction in inflammation and inactive disease, rehabilitative surgery can be considered. Orbital decompression surgery should be performed in patients with poor response or no response within 2 weeks. First, treatment initiation with IV corticosteroids is recommended [13]. However, owing to the complexity of the disease and the variability in its course and presentation, individualized management is required. Depending on disease activity and severity, our patients received either 500 mg methylprednisolone weekly for 6 weeks followed by 250 mg weekly for another 6 weeks [12], or 500 mg methylprednisolone daily on 3 consecutive days in accordance with EUGOGO [13].

The evaluation period of our patients was between 2006 and 2020. Medical and surgical management of patients with TED changed over time and advances were made to restore visual function and improve appearance and ocular motility. Regarding surgical therapy early strategies included lateral orbitotomy or transcranial decompression. At that time, limited understanding of the pathomechanism of the disease existed. By 2000, two- and three-wall decompression techniques had been introduced and were subsequently refined as the pathophysiology of TED became better understood [25]. Treatment has since shifted towards individualized, multidisciplinary management tailored to each patient’s needs. Medical therapy progressed from oral to IV glucocorticoids, which showed a better treatment response, and weekly and high-dose IV glucocorticoid regimens were established to further improve outcomes [11,12,13,22,26]. Medical treatment continues to evolve, most notably with the introduction of biologic agents. These evolving treatment regimens should be taken into account in future studies.

In our analysis we also looked at unilateral DON cases. Reported risk factors include higher age and also vascular disease, and patients with those risk factors showed reduced outcome after treatment [17,18]. In our analysis, VA was preserved in most patients with unilateral DON over the observation period, although a slightly higher proportion showed a decrease in VA compared with the overall cohort of patients with asymmetric TED. A total of 88% had a final VA of <0.3 logMAR. Diplopia was the most common presenting symptom, and upgaze restriction was the principal clinical finding. Most patients with unilateral DON underwent three-wall decompression, and the reduction in proptosis after orbital decompression and/or IV corticosteroids was 4 mm. A higher amount of patients received intravenous corticosteroids before orbital decompression surgery when compared to unilateral/asymmetric TED patients. Also, more patients needed rehabilitative surgery when compared to patients with asymmetric TED. Early detection of DON is essential for timely and adequate treatment. Ultrasonography and MRI/CT are therefore important for diagnostic evaluation and surgical planning. In unilateral presentations, imaging is usually performed promptly to exclude other conditions such as orbital tumours, which may lead to earlier diagnosis and treatment of DON and thus to favourable visual outcomes.

Limitations of this study include the retrospective nature and varying follow-up durations. In addition, no eye position photography or typical asymmetric CT images of the patients could be included. A further limitation that needs to be mentioned is the definition of asymmetric disease. A >2 mm difference in proptosis between the eyes was defined as asymmetric disease including patients with soft-tissue involvement in the contralateral eye. However, with this investigation, the different presenting features of patients with asymmetric TED and unilateral DON were shown. This cohort of patients represents patients who were treated with orbital decompression surgery. Further studies are necessary to evaluate a general population of patients with asymmetric TED.

## 5. Conclusions

The presenting features of asymmetric TED are heterogeneous and must be considered in patients with atypical disease. Most of our patients were treated with IV corticosteroids followed by orbital decompression, predominantly three-wall decompression. Visual function was preserved in a high proportion of patients, and exophthalmos was reduced by a mean of 4 mm. Individualized evaluation and treatment planning, combining corticosteroid therapy and decompression surgery, are essential in the management of asymmetric TED.

## Figures and Tables

**Table 1:** Characteristics of patients with asymmetric TED (*n* = 48).

	Percentage (Number)
**asymmetric TED**	50 (24)
**unilateral DON**	50 (24)
	
**Eye affected**	
right	38 (18)
left	62 (30)
**Sex**	
male	42 (20)
female	58 (28)
	
**Diagnosis**	
Basedow hyperthyroidism	77 (37)
Hashimoto thyroiditis	2 (1)
hypothyreosis	2 (1)
no known thyroid disease	19 (9)
	
**Treatment**	
History of radioactive iodine	33 (16)
History of thyroidectomy	24 (50)
Treatment with anti-thyroid drugs only	54 (26)

**Table 2:** Outcome of the patients with asymmetric TED.

Parameters	Number of Patients	Initial Presentation	Last Follow-Up	*p*-Value
				
**Visual acuity (logMAR)**	48	0.1 ± 0.5 *	0.1 ± 0.5 *	0.4
				
**Proptosis (in millimeters)**		23 ± 3 *	19 ± 3 *	*<0.01*
2-wall decompression surgery	13	24 ± 3 *	20 ± 3 *	*<0.01*
3-wall decompression surgery	35	23 ± 3 *	19 ± 3 *	*<0.01*
				
**Clinical activity score**	34	3 ± 2 *	1 ± 2 *	*p* > 0.05
				
**Intraocular eye pressure**	48	22 ± 6 *	20 ± 5 *	*p* > 0.05
				
**Width of the intrapalpebral fissure**	48	11 ± 2 *	10 ± 2 *	*p* > 0.05

* mean ± standard deviation.

**Table 3:** Characteristics of patients with unilateral DON at initial presentation (*n* = 24).

	Percentage (Number)
**Gender**	
female	50 (12)
male	50 (12)
**Diagnosis**	
Basedow hyperthyroidism	75 (18)
Hashimoto thyroiditis	13 (3)
Hyperthyroidism	4 (1)
none	8 (2)
**Thyroid specific medication**	
thiamazole	25 (6)
levothyroxine	17 (4)
levothyroxine-natrium	25 (6)
none	25 (6)
unknown	8 (2)

**Table 4:** Differences between patients with or without DON (*n* = 24 in each groups).

	Patients Without DON	Patients with DON Diagnosis	*p*-Value
**Initial visual acuity**	0.1 ± 0.5	0.1 ± 0.5	0.2
**Visual acuity at last follow-up**	0.1 ± 0.6	0.2 ± 0.5	0.8
			
**Proptosis initial**	23 ± 6	24 ± 3	0.7
**Proptosis last follow-up**	19 ± 3	19 ± 3	0.4
			
**Initial CAS**	2 ± 3	3 ± 2	0.6
**CAS at last follow-up**	0 ± 1	0 ± 1	0.4

## Data Availability

Data are not applicable for privacy.

## Abbreviations

The following abbreviations are used in this manuscript:

TED	Thyroid eye disease
DON	Dysthyroid eye disease
VA	Visual acuity
SNI	Superonasal muscle index
IV	Intravenous
